# Cytokines Stimulate the Release of Microvesicles from Myeloid Cells Independently from the P2X7 Receptor/Acid Sphingomyelinase Pathway

**DOI:** 10.3389/fimmu.2018.00204

**Published:** 2018-02-07

**Authors:** Federico Colombo, Mattia Bastoni, Annamaria Nigro, Paola Podini, Annamaria Finardi, Giacomo Casella, Menon Ramesh, Cinthia Farina, Claudia Verderio, Roberto Furlan

**Affiliations:** ^1^Department of Neuroscience and INSPE, San Raffaele Scientific Institute, Milano, Italy; ^2^CNR Institute of Neuroscience, Milan, Italy

**Keywords:** microvesicles, inflammation, cytokines, myeloid cells, multiple sclerosis, transcription, P2X7

## Abstract

Microvesicles (MVs) are membrane particles of 200–500 nm released by all cell types constitutively. MVs of myeloid origin are found increased in the cerebrospinal fluid (CSF) of patients suffering from neuroinflammatory disorders, although the factors triggering their production have never been defined. Here, we report that both pro- and anti-inflammatory cytokines, specifically interferon-γ and interleukin-4, are equally able to stimulate the production of MVs from microglia cells and monocytes. Additionally, we found this process to be independent from the best characterized molecular pathway so far described for membrane shedding, which is centered on the purinergic receptor P2X7, whose activation by high concentrations of extracellular ATP (exATP) results in membrane blebbing operated by the secreted enzyme acid sphingomyelinase (ASMase). Moreover, a potent inhibitor of ASMase, injected in a mouse model of multiple sclerosis, failed to reduce the number of MVs in their CSF. This suggests that cytokines, rather than exATP, may exert a long-term control of MV production in the context of chronic inflammation, where both pro- and anti-inflammatory factors play coordinated roles.

## Introduction

Extracellular vesicles (EVs) are microparticles produced by all cell types in physiological and pathological conditions. Currently, two main types of EVs have been described, microvesicles (MVs) and exosomes, whose differences reside in the dimension as well as in the mechanisms underlying their generation. MVs are particles of 150–500 nm budding from the plasma membrane, whereas exosomes are nanovesicles of 20–100 nm formed intracellularly. Actually, the molecular mechanisms participating in EV biogenesis are known only in part. As far as their functions are concerned, two main hypotheses have been proposed: (i) EVs may act as a system for cell-to-cell communication by shuttling active biomolecules among cells, (ii) they could serve as vehicles for discharging damaged molecules (lipids and proteins) out of the cell, lightening the ubiquitin-proteasomal system as well as lysosomes in case of overload. In recent years, the interest for EVs increased exponentially starting from the observation that in many different diseases their number is significantly increased in the body fluids of patients (1–5). For this reason, EVs are currently under intense investigation for a possible employment in the clinical practice as prognostic biomarkers. Recently, our group reported MVs, derived from myeloid cells (monocytes, macrophages, and microglia), to be significantly upregulated in the cerebrospinal fluid (CSF) of patients with neuroinflammatory disorders, in particular of those suffering from the most severe forms of multiple sclerosis (6). The stimuli responsible for the production of such MVs from myeloid cells are not known, although the role of extracellular ATP (exATP) and its receptor P2X7 has been widely proven, albeit only *in vitro*; whether exATP recapitulates this effect also *in vivo* needs to be defined.

Here, we describe the ability of both pro-and anti-inflammatory cytokines, the most represented class of soluble molecules orchestrating inflammatory processes, to enhance the release of MVs from myeloid cells. Additionally, we found this process to be unrelated to that induced by exATP through the activation of its receptor P2X7, but strictly dependent on transcription. Moreover, by using the mouse model of multiple sclerosis, the experimental autoimmune encephalomyelitis (EAE), we found that injection of imipramine, a well-established inhibitor of MV release mediated by exATP (7), did not affect the number of myeloid MVs in the CSF of such mice, with respect to controls. Overall, these findings might suggest the existence of a pathway stimulated by cytokines, but alternative to that centered on the exATP/P2X7 signaling axis, which could be involved in the release of myeloid MVs during inflammatory conditions.

## Materials and Methods

### Cell Cultures and Transfections

CHME-5 and BV2 cells were cultured in Dulbecco Modified Eagle’s Medium (DMEM, Gibco) supplemented with 10% fetal bovine serum, penicillin-streptomycin (100 U/ml) and 2 mM l-glutamine. Transfections were carried out by Lipofectamine LTX (Invitrogen) according to the manufacturer’s instructions; the p277.pCCLsin.hPGK plasmid encoding for farnesyl-GFP (f-EGFP) was provided by Prof. Luigi Naldini (Università Vita-Salute San Raffaele, Milano). Peripheral blood mononuclear cells were separated from whole blood by density gradient centrifugation (Ficoll-PaqueTM Plus, GE Healthcare) and CD14^+^ monocytes were purified by immunomagnetic beads [immunomagnetic MicroBeads (MACS^®^ Miltenyi Biotec)]. Monocytes were stimulated with cytokines 18 h after seeding. Blood samples came from three healthy donors recruited among the lab workers, who signed an appropriate informed consent. The study was approved by the local Ethical Committee.

### Antibodies and Reagents

The following antibodies were used: rabbit anti-P2X7 (Alomone Labs), mouse anti-flotillin-1 (BD Bioscience), and rabbit anti-Ki67 (Novocastra). Phalloidin-488 was used to stain F-actin; the monoclonal antibody against desmoyokin-AHNAK (dA) was a gift of Prof. Jacopo Meldolesi (Università Vita-Salute San Raffaele, Milano, Italy). Rabbit anti-Alix (Millipore), goat anti-CD63 (Biorbyt), mouse anti-COX-IV (Cell Signaling Technology), rabbit anti-cleaved caspase-3 (Cell Signaling Technology), β-actin (Sigma). Oxidized ATP (oxATP), brilliant blue G (BBG), probenecid, imipramine, siramesine and actinomycin D (actD) and ethidium bromide were purchased from Sigma-Aldrich; ARL67156 and SR11302 were from Tocris. ^10^Panx was from Innovagen and 11R-VIVIT from Calbiochem. WP631 was a gift of Dr. Cinthia Farina (San Raffaele Scientific Institute, Milano, Italy).

### Cell Treatments

Interleukin-4 (IL-4), gamma-interferon (IFN-γ), interleukin-5 (IL-5), interleukin-13 (IL-13), interleukin-23 (IL-23), interleukin-27 (IL-27), transforming growth factor-beta (TGF-β), interleukin-6 (IL-6, R&D), and tumor necrosis factor-α (TNF-α Peprotech) were used at a final concentration of 20 ng ml^−1^, for 24 h (unless otherwise stated). ATP was used at 1 mM for 5–30 min. In all experiments, pharmacological inhibitors were added to the cell medium, composed by DMEM supplemented with vesicle-depleted serum, 1 h before the addition of cytokines and 24 h before that of ATP, and maintained throughout the treatments: in this way the effects of the inhibitors on cytokine and ATP treatments can be compared.

### Ethidium Uptake

CHME-5 cells were washed twice with Krebs-Ringer Hepes (KRH) solution (125 mM NaCl, 5 mM KCl, 1 mM MgSO_4_, 1 mM KH_2_PO_4_, 25 mM HEPES, 1 mM CaCl_2_, 6 mM glucose) and maintained for 15 min in the presence of ethidium bromide (15 µM, Sigma-Aldrich). ATP (1 mM) was added and the cells were imaged by using an Axiovert A1 microscope (Karl Zeiss) equipped with an Axio Cam ICm1 and a HPX 120 V lamp. Cells treated with ethidium but not with ATP were used as a control.

### Ca^2+^ Imaging

Ca^2+^ influx was assessed by imaging using Fluo-4AM as a Ca^2+^ indicator; briefly, CHME-5 cells were washed two times with KRH solution, then incubated in the dark with Fluo-4AM (4 µM in KRH) at room temperature for 45 min. Pluronic acid (20% w/v) was used for keeping Fluo-4AM in solution. Afterward, cells were washed two times in KRH and incubated for 20 min at 37°C for allowing the complete de-esterification of the indicator. After an additional wash in KRH, cells were treated or not with ATP (1 mM) and imaged under an Axiovert A1 microscope (Karl Zeiss). Untreated cells were used as a control for determining the baseline fluorescence of Fura-4AM.

### Quantification of exATP

Extracellular ATP was quantified by using the luminescent ATP detection assay from Abcam; the assay quantifies the amount of light emitted by luciferin when oxidized by luciferase in the presence of ATP and oxygen. Cells were plated into a 96-well plate and stimulated for different times with IL-4 or IFN-γ in triplicates, in complete medium (100 µl); for each time point untreated wells were used. At the end of the treatments the plate was centrifuged at 1,000 rpm for 5 min in order to pellet floating cells and 80 out of 100 µl were transferred in a black-plate for fluorimetric readings. ATP standards were loaded on the same plate as references. The following steps were performed according to the manufacturer’s instructions; light emission was quantified with Victor3 (Wallac).

### MV Purification by Differential Ultracentrifugation

Microvesicles were purified from the cell media by using a standardized protocol with slight modifications (7): conditioned cell media were collected and centrifuged for 10 min at 300 *g* for removing floating cells and debris. The resulting supernatants were further cleared through a 5-µm syringe-filter (Millex, Millipore), then ultracentrifuged at 10,000 *g* for 30 min to pellet MVs. The pellets obtained were resuspended in lysis buffer with protease inhibitor, PBS or fixative depending on the specific aim. As serum contains high levels of MVs, cells were cultured in Optimem or in DMEM supplemented with serum depleted of MVs by an overnight ultracentrifugation (at 110,000 *g*) as described in Shelke et al., (8).

### MV Quantification by Flow Cytometry

Conditioned cell media were centrifuged at 300 *g* (for 10 min) and filtered through a 5-µm syringe-filter (Millex, Millipore) in order to remove cell debris and apoptotic bodies. The resulting supernatants were stained with the FITC-conjugated isolectine B4 from *Bandeiraea simplicifolia* (Sigma-Aldrich), previously centrifuged at 13,000 *g* for 30 min for discarding aggregates. Calibration beads (BioCytex) of known dimensions (from 0.1 to 0.9 µm) were used to define the gate into which IB4^+^ events can be considered as *bona fide* MVs. Samples were acquired at Accuri C6 (BD Biosciences).

### MV Quantification by Tuneable Resistive Pulse Sensing (TRPS)

Purified MVs were resuspended in filtered PBS and an aliquot of such suspension (40 µl) was loaded into the nanopore (NP200) previously activated by multiple washes with PBS. The recordings were performed with qNano™ (Izon) using a voltage-pressure protocol, according the manufacturer’s instructions. Calibration particles (cpc200b, Izon) were used to define the dimensional range of the measured MVs.

### Fluorescence Microscopy

Cells were fixed with 4% para-formaldehyde (10 min at 4°C), quenched with 0.1 M glycine and processed for indirect immunofluorescence. Images were collected by using a widefield microscope (Olympus IX70) coupled to the DeltaVision deconvolution system (GE Healthcare).

For Ki67 assay cells were seeded on 96-well plates (Corning) and stained; images were blindly acquired by InCell Analyzer 1000 (GE Healthcare) and analyzed with the ImageJ software.

### Live-Cell Imaging

f-GFP transfected cells, sparsely grown on glass coverslips, were washed in PBS and maintained in Optimem (Gibco). After few minutes of recording, ATP (1 mM) was added to the medium for inducing the formation of MVs. All the frames collected were deconvoluted and assembled as a movie with ImageJ software.

### Transmission Electron Microscopy (TEM) for Cells and MVs

After brief treatment with ATP, CHME-5 cell monolayers were detached and sedimented by centrifugation (1,000 rpm; 5 min); the pellets were then fixed with 4% paraformaldheyde-2% glutaraldheyde in PBS (for 30 min), post-fixed with 1% OsO_4_ (1 h), then washed and embedded in Epon. Conventional thin sections were collected on uncoated grids, stained with uranyl acetate and lead citrate, and examined in a Leo 912 electron microscope (Zeiss).

Microvesicles were observed at TEM after being contrasted by negative staining: cell media were fractionated by differential ultracentrifugation, the resulting pellets resuspended in 20 µl of PBS and adsorbed to 400-mesh formvar/carbon coated grid for 10 min at RT. Adherent vesicles were stained with uranlyl acetate and immediately observed at the electron microscope.

### Scanning Electron Microscopy (SEM)

For SEM 1 mM ATP was added to CHME-5 cells sparsely grown on 10-mm glass coverslips; the fixation step was performed with a solution composed by 4% paraformaldheyde-2% glutaraldheyde (in PBS) at RT. Samples were post-fixed in 1% OsO_4_, dehydrated in ethanol, critical-point dried and sputter-coated with gold palladium for 50 s. Images were collected with a Leica S420 scanning electron microscope.

### Western Blot Analyses of Cells and MVs

Purified MVs were resuspended in lysis buffer supplemented with a protease inhibitor cocktail (Sigma-Aldrich). Protein concentrations were measured with BCA (Micro BCA, Pierce). 3–8 µg of MV extracts were diluted with Laemmli buffer and loaded into 8–14% polyacrylamide gels.

### RT-PCR Analysis

Cells were lysed in Trizol (Invitrogen) and RNA was purified according to the manufacturer’s instructions; residual DNA was removed by DNase treatment at 37°C for 30 min. cDNA synthesis from 5 µg of total RNA was performed using Ready-To-Go You-Prime First-Strand Beads (Amersham) and Random Hexamer (New England Biolabs) according to the manufacturer’s instructions. The levels of P2X7 and GAPDH mRNA was measured by real time RT-PCR (Applied Biosystem); P2X7 (Hs00175721_m1), GAPDH (Hs99999905_m1). The 2^−ΔΔCT^ method was used to calculate relative changes in gene expression as previously described (9).

### IL-1β Quantification by ELISA

CHME-5 cells were seeded at high density on 10-cm Petri dishes and stimulated with IFN-γ, IL-4 (20 ng ml^−1^, 24 h), lipopolysaccaride (LPS) (100 ng ml^−1^, 24 h), or LPS (100 ng ml^−1^, 24 h) + ATP (1 mM, 30 min). Untreated cells were used as negative control. Cell supernatants were then collected and processed according to the above protocol of differential ultracentrifugation. The content of IL-1β in MVs was assessed by using the human IL-1β/IL-1F2 DuoSet ELISA Kit (R&D), according to the manufacturer’s instructions.

### Experimental Autoimmune Encephalomyelitis

Experimental autoimmune encephalomyelitis were induced into C57/B6 mice as previously described (9). All procedures involving animals were performed according to the animal protocol guidelines prescribed by the Institutional Animal Care and Use Committee authorization no. 643 at San Raffaele Scientific Institute (Milan, Italy). Imipramine (20 mg kg^−1^, Sigma) was injected into the peritoneum on the day of clinical onset; control mice were injected with the saline solution in which imipramine was dissolved. Injections were repeated every day and mice were weighed and scored for clinical signs daily up to the day of culling. Clinical assessment of EAE was performed according to the following scoring criteria: 0 = healthy; 1 = limp tail; 2 = ataxia and/or paresis of hindlimbs; 3 = paralysis of hindlimbs and/or paresis of forelimbs; 4 = tetraparalysis; and 5 = moribund or death.

Before the sacrifice mice were deeply anesthetized for CSF collection performed with a glass capillary introduced into the cisterna magna; PBS-diluted CSF samples were stained with FITC-conjugated IB-4 and the MV content analyzed by flow cytometry and TRPS.

### Transcriptomic Analysis

RNA from unstimulated and IFN-γ + LPS or IL-4-treated Pec macrophages, prepared as described (10), were amplified according to Illumina TotalPrep RNA Amplification Kit protocol (Illumina). MouseWG-6 v2 arrays (Illumina) were employed for direct hybridization, according to the manufacturer’s instructions. The raw data were exported using GenomeStudio software (Illumina) and processed further in Bioconductor. The Minimum Information about a Microarray Experiment compliant microarray data have been deposited in the European Bioinformatics Institute ArrayExpress database (accession number: E-MTAB-6416). Probes with a detection *p*-value lower than 0.05 in at least one sample in a given group were filtered out.

For the differential expression analysis we selected those probes passing the *p*-value threshold of 0.05 and with a minimum fold change of 1.4 under cell stimulation. Further we focused on those differentially expressed probes with a minimum expression intensity of 500 in a given group so to select for transcripts already expressed also in the unstimulated cells. Genecodis (11) was used for transcription factor (TF) enrichment analysis and selected those terms passing FDR-corrected *p*-value threshold of 0.05 and containing at least three genes.

### Statistical Analyses

All statistical analyses were performed with the Prism software (Graph Pad). The tests applied are specified in the figure legends.

## Results

### Imipramine Does Not Reduce MV Production in EAE Mice

**Figure 1:**
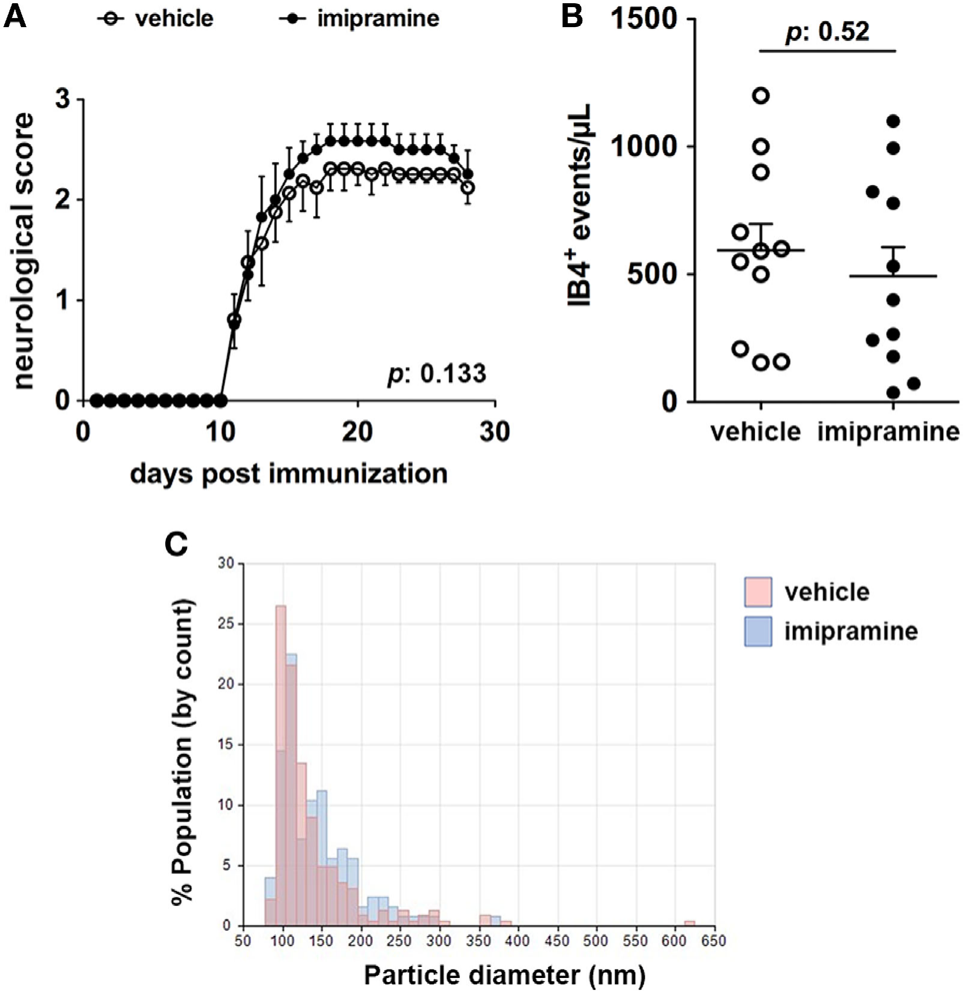
Imipramine does neither modulate the clinical score of experimental autoimmune encephalomyelitis (EAE) mice nor reduce the amount of IB4^+^ microvesicles (MVs) in the cerebrospinal fluid (CSF). **(A)** Clinical score of EAE mice daily injected with 20 mg kg^−1^ imipramine (11 mice) or vehicle (11 mice), starting after disease onset (12-day post immunization). Each dot represents the mean score ± SEM of the 11 mice per condition used (open circles: vehicle treated; black dots: imipramine treated). For statistical analysis Mann–Whitney test was applied. No difference in terms of clinical score can be observed between the two treatments (*p*: 0.133). **(B)** CSF was collected from each mouse and the content of IB4^+^ MVs was measured by flow-cytometry. Statistical analysis, performed with unpaired Student’s *t*-test (two tails), reveals no significant difference in terms of MV number between the two conditions (*p*: 0.52). The graphs in **(A,B)** show one of representative of two independent experiments. **(C)** Pooled CSF samples were measured by tuneable resistive pulse sensing. The histograms display counts (*y* axis) and estimated size distribution (*x* axis) of MVs. The graph is representative of three measurements from two independent experiments. There are no differences in CSF MVs size between imipramine and untreated mice.

Imipramine, a triciclic antidepressant drug, has been shown to be effective *in vitro* in reducing the release of MVs mediated by exATP (7). In order to assess the role of exATP in the release of MVs during inflammation of the nervous system, EAE mice were treated with imipramine and the disease course was daily monitored with respect to that of vehicle-injected animals. We chose the dose of imipramine which efficiently exerts its neurotropic (anti-depressive) effects in mice once injected intraperitoneally (12). At the end of the experiment, CSF was collected from each mouse and the number of myeloid MVs was quantified by flow-cytometry. Interestingly, imipramine did modify neither the clinical evolution of the disease (Figure 1A) nor the amount of myeloid cell-derived MVs in the CSF of treated versus vehicle-injected controls (Figures 1B,C). These results suggest that exATP probably has a minor role in the generation and/or maintaining the MV production elevated during chronic neuroinflammation.

### CHME-5 Cells Are a Suitable Tool to Study Regulation of MV Release from Microglia

**Figure 2:**
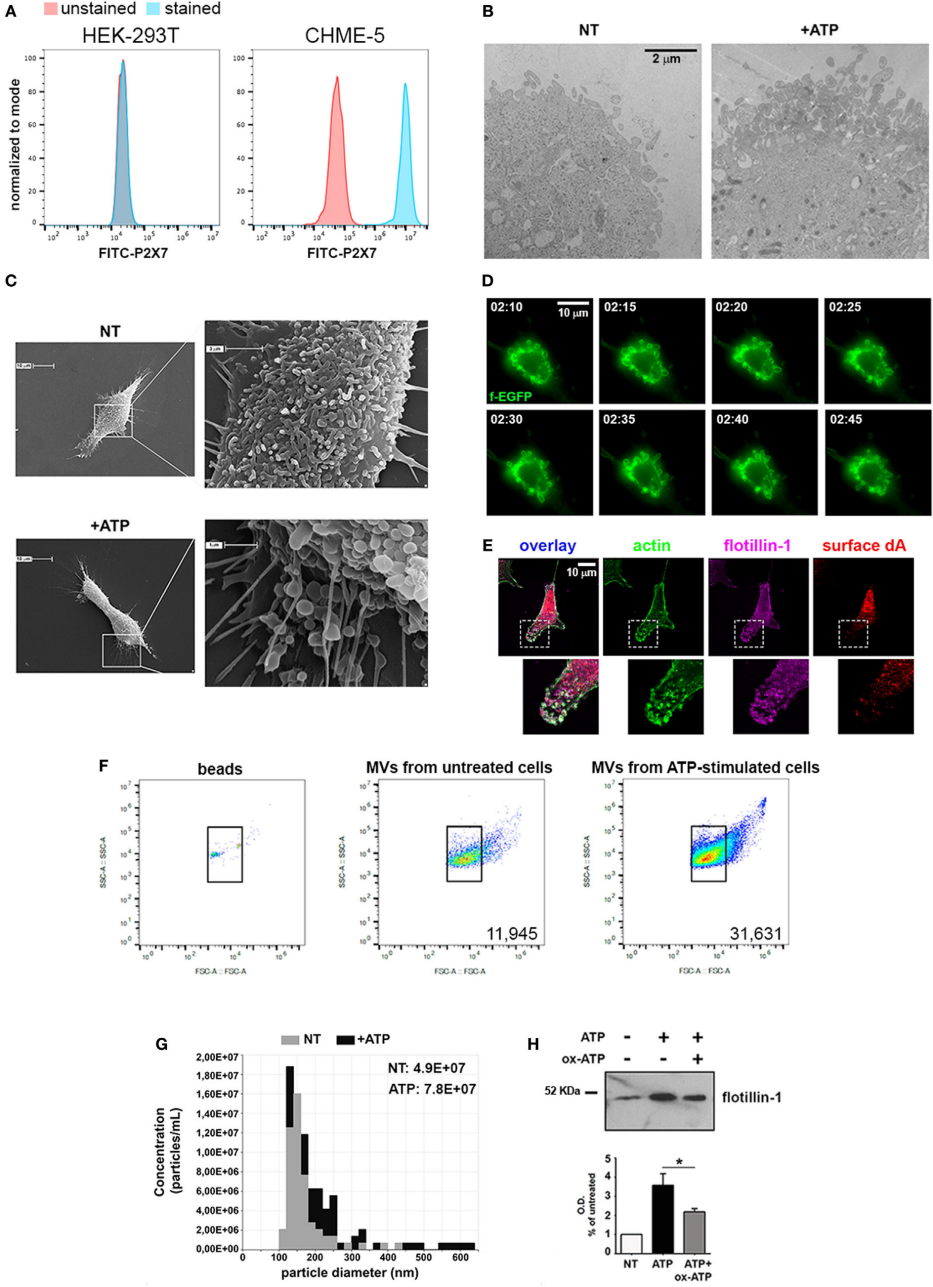
CHME-5 cells produce microvesicles (MVs) in response to ATP. **(A)** Analysis of the surface expression of P2X7-R by flow-cytometry in HEK-293T (left panel) and CHME-5 cells (right panel). Unstained cells (red curves) were used as negative control; stained HEK-293T and CHME-5 cells are represented as blue curves. **(B)** Electron micrographs showing the plasma membrane of not treated (NT, left panel) and ATP-treated (+ATP, right panel) CHME-5 cells. Multiple round-shaped structures budding from the plasma membrane appeared mainly at the cell surface of ATP-treated cells. The images are representative of two experiments, in which at least 15 fields per condition have been analyzed. The scale bar on the right, valid for both panels, is 2 µm. **(C)** Scanning electron micrographs of representative CHME-5 cells, untreated (NT, upper panels) or stimulated with 1 mM ATP for 7 min (+ATP, lower panels) are shown. Insets magnifications are shown in right panels. These images are representative of 20 randomly selected fields per condition. **(D)** Snapshots taken from live-cell recordings of farnesylated-GFP transfected cells exposed to ATP (1 mM, 7 min) are shown; the scale bar at the upper right is 10 µm. The arrows indicate the dynamics of a portion of the plasma membrane during the blebbing/shedding process. The original Video S1 is enclosed into Supplementary Material. **(E)** A representative cell stained with antibodies against actin (green), surface desmoyokin-AHNAK (dA, red), and flotillin-1 (magenta); 6× magnifications of nascent vesicles are shown in the insets. Scale bar at the upper left is 10 µm. **(F)** The set up for flow cytometry quantification of MVs is shown: (left panel) FITC-conjugated beads of known dimensions (0.3–0.9 µm) were used as reference for gating. *Bona fide* MVs would correspond to the IB4^+^ events comprised into the gate determined by beads positioning inside the plot. Untreated versus ATP-stimulated CHME-5 cells were used as sources of MVs to test the sensitivity of this set up (middle and right panels, respectively), as well as that of tuneable resistive pulse sensing **(G)** and western blotting for flotillin-1 **(H)**; in the latter a control condition with oxidized ATP (oxATP), a P2X7 inhibitor, was included. Full-length blots are presented in Figure S4 in Supplementary Material.

Given these premises, we specifically wondered whether cytokines, which orchestrate many different aspects of the inflammatory process, could have a major role in MV release with respect to exATP. In order to clarify this point we used the human embryonic microglia cell line (CHME-5). Cell lines are unavoidable due to the fact that MVs are fragile structures scarcely produced by primary cell cultures; for any analyses, in contrast, huge amounts are necessary. In order to validate the model we checked the ability of CHME-5 cells to release MVs upon ATP treatment. The expression of the P2X7, the only receptor for extracellular purines so far related to membrane shedding (13), has been analyzed by flow-cytometry with respect to that of HEK-293T, a human cell line that does not express the receptor (14); Figure 2A shows that CHME-5 cells carry the P2X7 on their surface. Additionally, the expression of the receptor has been validated by RT-PCR and immunoblotting as well as by functional assays, i.e., ethidium uptake and calcium imaging (Figures S1A–D in Supplementary Material).

Accordingly, by TEM we demonstrated that a brief stimulation with ATP (1 mM, 5–7 min) led to the formation of multiple bleb-like structures at the plasma membrane, a morphological change typically induced by ATP (Figure 2B); the identity of such structures was confirmed by SEM (Figure 2C). Moreover, the kinetics of the process was studied by live-cell imaging of CHME-5 cells transfected with a membrane-bound form of GFP (farnesyl-GFP): multiple and motile blebs appeared at the plasma membrane 2 min after the addition of ATP (Figure 2D). As MVs are expected to retain a typical set of proteins, their presence on the multiple bleb-like structures that can be observed also by conventional fluorescence microscopy was tested. Figure 2E shows that such structures were stained by FITC-conjugated phalloidin (which labels F-actin) and by antibodies against the raft-resident proteins flotillin-1 and desmoyokin-AHNAK (d/A), both found already associated to MVs (15).

Purification of MVs from ATP-treated cell supernatants was achieved by a standardized protocol based on differential centrifugations of conditioned cell media (Figure S2A in Supplementary Material): floating cells and membrane remnants were removed by a brief low-speed centrifugation and filtration, followed by a step of ultracentrifugation (10,000 *g* for 30 min). For electron microscopy analyses, the resulting pellet was resuspended in PBS and contrasted by uranyl acetate; several particles with a mean diameter of 200 nm appeared in this fraction (Figure S2B in Supplementary Material). Analogous indications came from the analyses of the pellets performed with dynamic light scattering and TRPS measurements (Figures S2C,D in Supplementary Material). The content of MVs with respect to that of exosomes and parental cells was analyzed by western blotting (Figure S2E in Supplementary Material). Flotillin-1 was equally present in both particle fractions at variance with Alix, more abundant in the exosomal rather than the MV fraction, as expected (16). The mitochondrial-resident protein COX-IV was absent from both exosomes and MVs, further confirming that the preparation was devoid of cells.

Flow cytometry, TRPS and western blotting were employed for MV quantification. For cytofluorimetric measurement calibration beads of known dimensions (0.1–0.9 µm) were used for defining the area of the plot into which MVs were expected to be comprised (Figure 2F, left panel). As a proof of concept, we tested whether the stereotyped effects of exATP on MV production could be recorded by using this set up. As reported in Figure 2F (middle and right panels), the number of MVs produced by ATP-stimulated cells is higher than that of MVs collected from untreated cells. Similar results were obtained by TRPS as well as by immunoblotting of flotillin-1 (Figures 2G,H, respectively). Overall, this set of experiments shows that CHME-5 cells express a functional P2X7 receptor that mediates the release of MVs upon exATP treatments.

### Both IFN-γ and IL-4 Enhance MV Production

**Figure 3:**
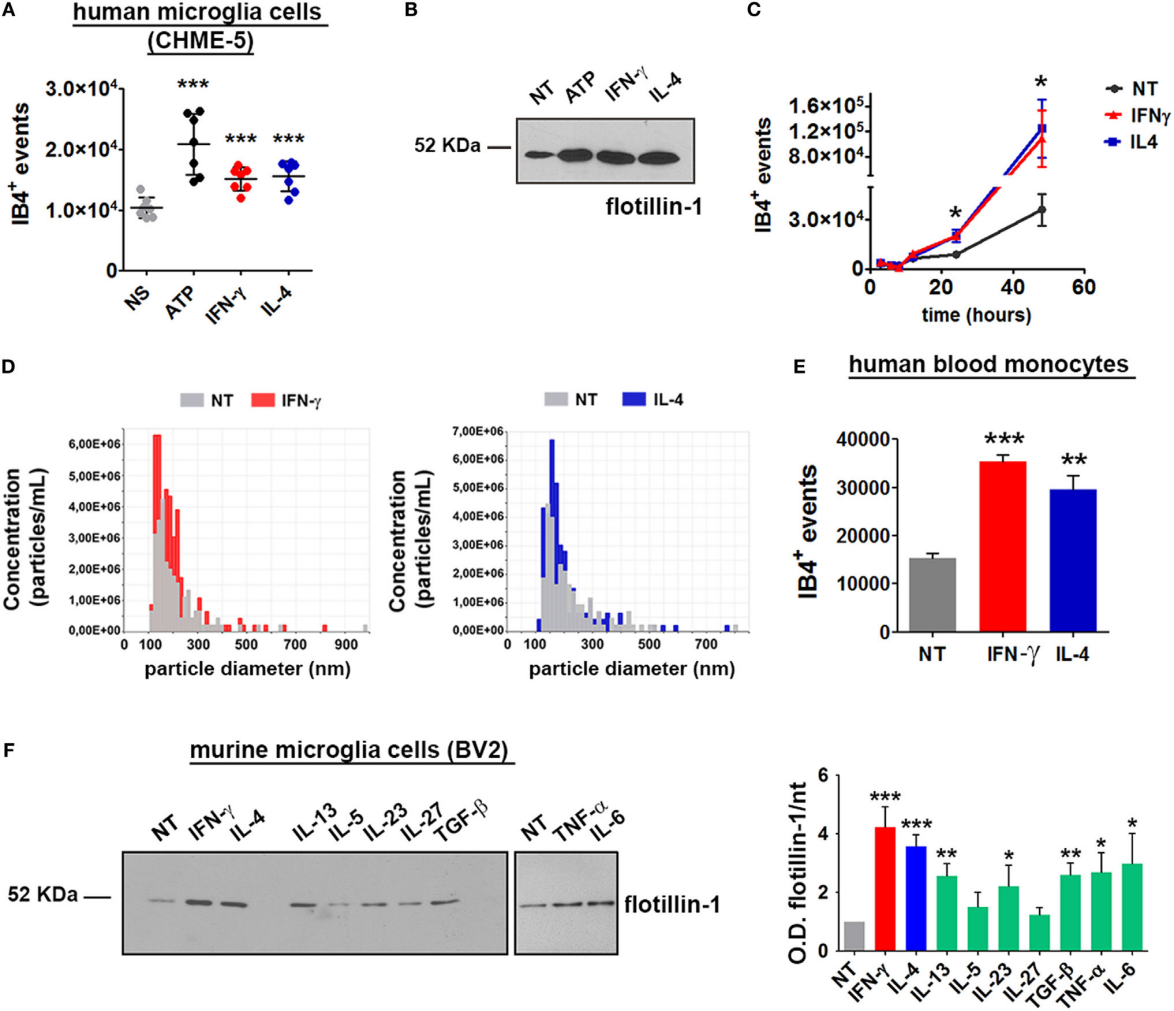
Interferon (IFN)-γ and interleukin-4 (IL-4) induce the release of microvesicles (MVs) from CHME-5, BV2 cells and human blood monocytes. MV release after cytokine (20 ng ml^−1^, 24 h) or ATP treatment (1 mM, 30 min), compared to untreated controls, was measured by **(A)** flow cytometry (mean ± SEM, seven independent experiments) or **(B)** western blotting for flotillin-1 (three independent experiments). For statistical analyses of flow cytometric measurements, one-way Anova plus Dunnett’s *post hoc* test were applied (***p: 0.0002; *p*: 0.0004; *p*: 0.0007, respectively). **(C)** Time-course analysis of MVs released after treatment with IFN-γ or IL-4 (20 ng ml^−1^, 3–48 h) quantified by flow cytometry (mean ± SEM, three independent experiments). For statistical analysis one-way Anova plus Dunnett’s *post hoc* test were applied (*p: 0.0199; *p*: 0.0479, respectively). **(D)** Tuneable resistive pulse sensing measurements of MVs released by cytokine-treated versus untreated CHME-5 cells are shown (three independent experiments). The release of MVs after IFN-γ and IL-4 treatments (20 ng ml^−1^, 24 h) was also analyzed using human blood monocytes **(E)** and BV2 cells **(F)**. MV release from BV2 cells was also tested by western blotting for flotillin-1 [**(F)**, left panel] after treatment with IFN-γ, IL-4, IL-13, interleukin-5 (IL-5), IL-23, IL-27, TGF-β, tumor necrosis factor-α (TNF-α), and IL-6 (20 ng ml^−1^, 24 h), as compared to untreated cells. The densitometric quantification of western blot is shown in the graph on the right. One-way Anova plus Dunnett’s *post hoc* test were applied [panel **(E)** ***p: 0.0003/**p: 0.081, panel **(F)** ***p: 0.0003 and 0.0004, respectively, **p: 0.0034 and 0.0030, respectively, *p: 0.0447]. In **(E,F)**, one representative of three independent experiments are shown (mean ± SEM). Full-length blots are presented in Figure S4 in Supplementary Material.

Having set up a proper system to analyze MV release from myeloid cells, the role of cytokines in MV production was tested: cells were treated for 24 h with IFN-γ or IL-4 and the content of MV in the supernatants was assessed by flow cytometry and immunoblotting. Of note, both cytokines significantly increased the amount of MVs released by CHME-5 cells compared to the untreated controls (*p*: 0.0004 and *p*: 0.0007, respectively) (Figures 3A,B). Moreover, time-course experiments revealed that, before 24 h, there was no difference between unstimulated and cytokine-treated cells, whereas from 24 to 48 h, the cytokine-mediated release of MVs was significantly increased (*p*: 0.0199 and *p*: 0.0479) (Figure 3C). TRPS confirmed the ability of both cytokines to enhance MV production from CHME-5 cells and also reported that such vesicles, in terms of dimension, are undistinguishable with respect to those produced by untreated cells (Figure 3D).

In order to verify whether the cytokine-mediated MV release is peculiar of this cell line or, alternatively, represents a general phenomenon, the same experiments were carried out by using the murine microglia cell line BV2. Long exposures (24 h) to IFN-γ and IL-4 enhanced the release of MVs also from human blood monocytes (*p*: 0.0003 and *p*: 0.0081) (Figure 3E). The effect of different cytokines on the release of MVs was tested by western blotting: similarly to CHME-5, BV2 cells respond to IFN-γ and IL-4 by releasing MVs. Also IL-13, IL-23, TGF-β, TNF-α, and IL-6 were found to share this property; on the other hand, IL-5 and IL-27 were not effective (Figure 3F). Previous reports had already demonstrated a strong proliferative effect of IFN-γ and IL-4 on T (17–19) and B lymphocytes (20); in contrast, the contribution of the two cytokines in macrophage cell expansion is contradictory, mainly as far as IL-4 is concerned. IFN-γ potently reduces myeloid cell proliferation (21) and protects cells from apoptosis by inducing p21-waf1 (22). p21-waf1 induction and cell cycle arrest have also been reported in IL-4-treated macrophages (23); however, an opposite observation has also been published (24). Therefore, we checked whether IFN-γ and IL-4 increase the number of microglia cells by accelerating cell cycle progression: in this case, the high levels of MVs induced by such stimuli would be simply due to a cell number higher than that of unstimulated controls. Upon IFN-γ or IL-4 treatment (for 12 and 24 h), microglia cells (BV2 and CHME-5) were stained with Ki67, a protein expressed in the nucleus of proliferating cells, and with DAPI for nuclei labeling. Cells were imaged and the fraction of Ki67-positive nuclei, as well as the total cell number, was calculated. Figure S3 in Supplementary Material shows that, at both 12 and 24 h, the percentage of proliferating (Ki67^+^) IFN-γ stimulated cells is significantly lower than that of IL-4 treated or untreated controls. The total cell number decreased after both IFN-γ and IL-4 treatment, confirming the observation previously reported by others (21–23). Therefore, the higher amounts of MV released by cytokines are not due to increased cell proliferation. Apoptosis has been excluded by MTT assays performed on cells treated with IFN-γ or IL-4 for 24 h (Figures S3A–F in Supplementary Material). Moreover, by western blotting we cannot detect the activated form of caspase-3 in the cells treated with ATP or in those exposed to cytokines (Figure S3G in Supplementary Material). IFN-γ and IL-4 trigger the production of MVs from myeloid cells in overlapping amounts and with similar kinetics but, as expected from our previous observations (10), with a different molecular signature. In the context of inflammation, one of the best characterized cargo of MVs is IL-1β, a cytokine with strong pro-inflammatory effects. It has been already described the ability of exATP to sustain the release of MVs loaded with IL-1β from microglia cells primed with LPS (25), a potent trigger of inflammation. Hence, we wondered whether MVs deriving from IFN-γ and IL-4 stimulation are characterized by different levels of IL-1-β.

We measured by ELISA the levels of IL-1β in the MV fraction of CHME-5 cell medium, after treatment with LPS, IFN-γ, and IL-4. As expected, cells treated with LPS for 24 h release IL-1β associated to MVs both in the presence or absence of brief ATP stimulation. However, we could not detect IL-1β in MVs released after IL-4 and IFN-γ treatments (Figure S3H in Supplementary Material). This result is in line with the anti-inflammatory properties of IL-4 in this context and confirms previous evidence reporting the inability of IFN-γ to promote the production of IL-1β in the absence of LPS (26).

### Enhanced MV Production from Cytokine-Treated Microglia Does Not Involve P2X7 Activation

**Figure 4:**
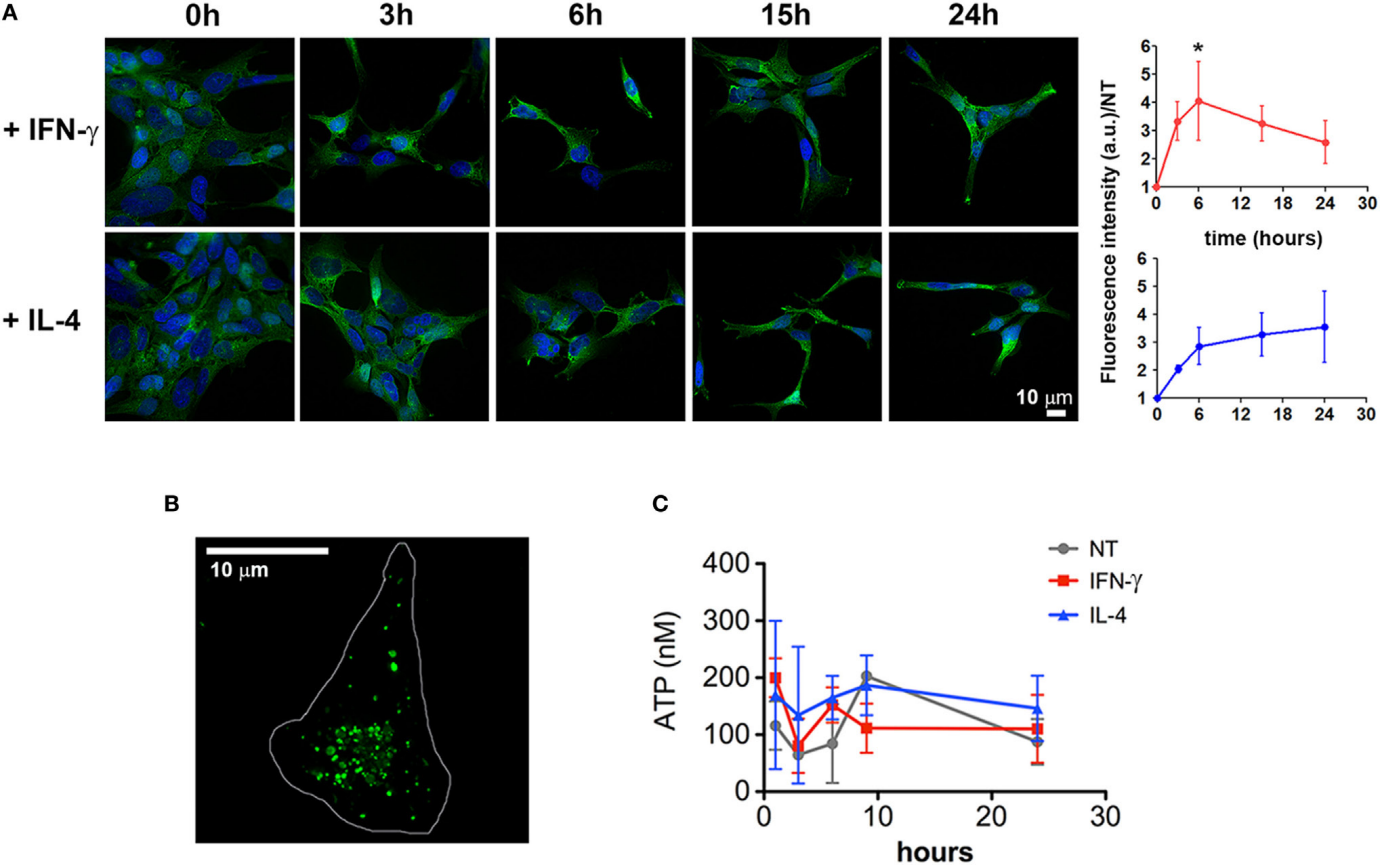
Cytokines induce a slight upregulation of P2X7 receptor. **(A)** CHME-5 cells were treated for 3, 6, 15, and 24 h with interferon (IFN)-γ or interleukin-4 (IL-4) (20 ng ml^−1^) and stained for P2X7; the normalized expression levels, calculated from four independent experiments (mean ± SEM), are reported on the graphs at the right side of the figure. One-way Anova plus Dunnett’s *post hoc* test were used for statistical analysis (*p: 0.0361). A significant increase of the P2X7 levels was measured only after 6 h of IFN-γ treatments. The scale bar in the upper right side of the image corresponds to 10 µm. **(B)** ATP-containing granules labeled by quinacrine staining of live CHME-5 cells were imaged by widefield microscopy followed by image deconvolution. Scale bars: 10 µm. **(C)** Extracellular ATP (exATP) quantified by a luminescence assay; to reduce exATP degradation the ecto-ATPase inhibitor ARL67156 was maintained in the culture medium throughout the treatments. Data represent the mean ± SEM of three independent experiments. Student’s *t*-test was used for statistical analysis. No significant differences were found between controls and stimulated cells.

In order to characterize the molecular pathway/s underlying this newly described cytokine function, we tested the hypothesis that they could enhance the release of MVs by regulating the P2X7 signaling network, at any level. One possibility is that IFN-γ and IL-4 increase the expression of the receptor allowing the cells to be more sensitive to exATP. Time-course experiments show that, starting from 3 h of stimulation both cytokines increased the levels of P2X7, although significantly only at 6 h of IFN-γ treatment (*p*: 0.0361) (Figure 4A). Additionally, we checked whether this receptor upregulation is also paired to an increase in exATP concentrations, a condition that can be achieved by extensive cell death or by exocytosis of specific ATP-storing granules (27). As IFN-γ and IL-4 seem to be anti-rather than pro-apoptotic factors (at least at the times and concentrations used here), the first possibility was unlikely. Intracellularly, microglial cells accumulate ATP into specialized granules which can be visualized by acridine derivatives such as quinacrine (Figure 4B). In order to check whether cytokines promote ATP exocytosis, cell supernatants were collected and processed for measuring the concentrations of exATP. For preventing exATP degradation, treatments were performed in the presence of an ecto-ATPase inhibitor: no significant differences were observed among the conditions investigated, at any of the time points tested (Figure 4C). The average concentrations of exATP measured here fall within a nanomolar range, which is in line with those previously reported for experiments in which ecto-ATPase inhibitors have been employed (28).

**Figure 5:**
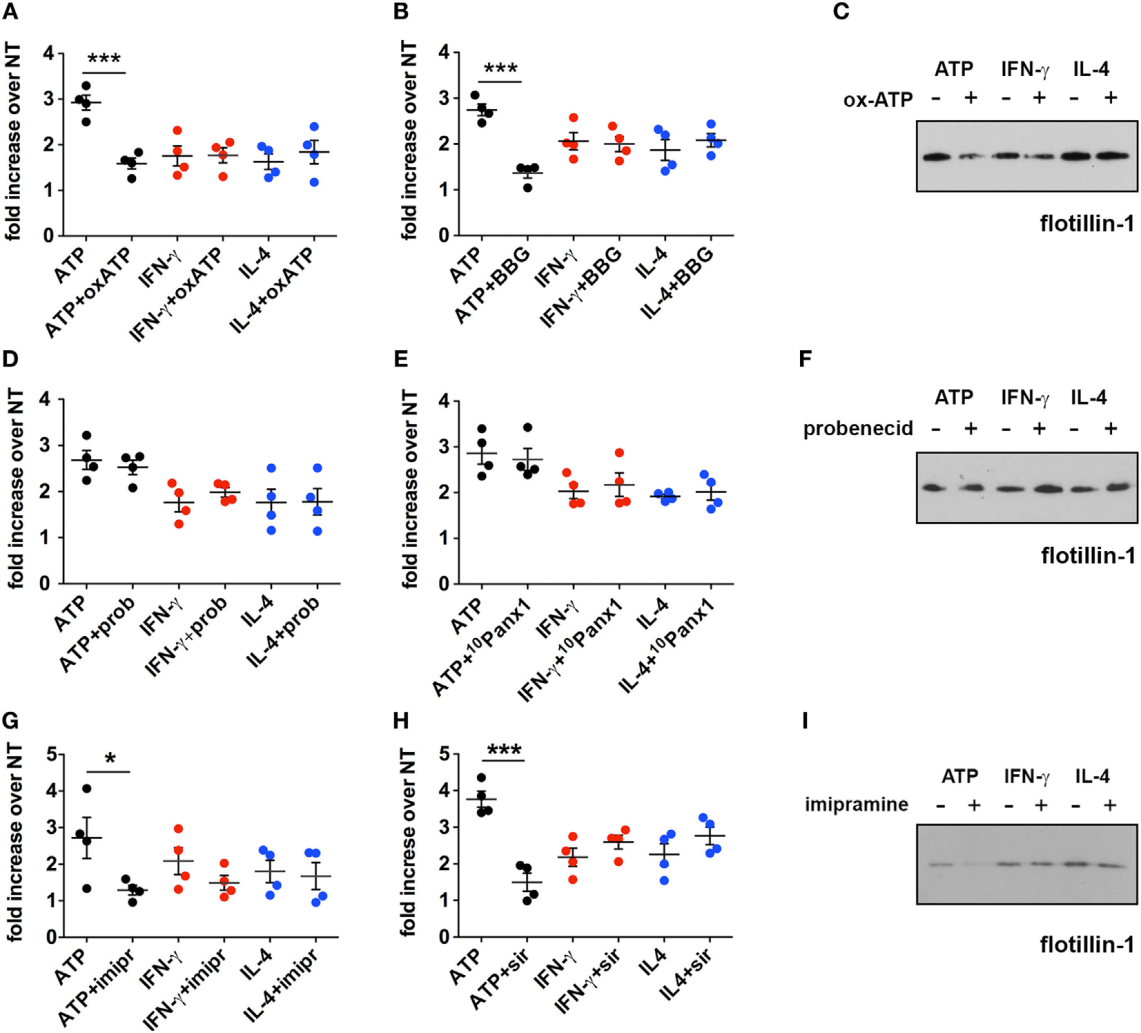
Inhibition of the P2X7 signaling pathway does not affect the cytokine-induced release of microvesicles (MVs) from CHME-5 cells. Quantification of MV release by flow cytometry **(A,B,D,E,G,H)** and western blotting for flotillin-1 **(C,F,I)** in the presence or absence of multiple inhibitors of the P2X7 signaling pathway. Data on the graphs are represented as fold increase compared to the untreated controls (mean ± SEM of four independent experiments). **(A–C)** Cells were treated with cytokines (20 ng ml^−1^) for 24 h in the presence or absence of oxidized ATP (oxATP) (200 µM) **(A,C)** or brilliant blue G (BBG) (100 nM) **(B)**. ATP treatments (1 mM, 30 min) ± inhibitors (24 h pre-treatments) were also performed. MVs were then collected and analyzed by flow cytometry and western blot. Unpaired two-tailed Student’s *t*-test was used for statistical analyses (***p: 0.0006/*p*: 0.0002). No significant difference of cytokine-induced MV release was found in the presence of oxATP and BBG that, however, reduce the MV release induced by ATP. **(D–F)** The same as in **(A–C)** but in the presence of two pannexin-1 inhibitors: probenecid (prob) (5 mM) **(D,F)** and the blocker peptide ^10^Panx1 (300 µM) **(E)**. No significant difference of cytokine- and ATP-induced MV release was found in the presence of probenecid and ^10^Panx1. **(G–I)** Imipramine (imipr) (10 µM) **(G,I)** and siramesine (sir) (8 µM) **(H)** were used as acid sphingomyelinase inhibitors. Unpaired two-tailed Student’s *t*-test was used for statistical analyses (*p: 0.0342/***p: 0.0004). No significant difference of cytokine-induced MV release was found in the presence of imipramine and siramesine, whereas they significantly reduce the release mediated by ATP. In all experiments inhibitors were applied 1 h before cytokine treatment, or for 24 h before ATP treatment, and maintained thereafter, to allow comparison of the effects of the inhibitors on ATP and cytokine-mediated MV production. All the western blots shown are representative of two independent experiments. Full-length blots are presented in Figure S4 in Supplementary Material.

Further experiments to establish the contribution of the P2X7 receptor in cytokine-mediated MV release were carried out by the use of specific P2X7 antagonists, oxATP (29) and BBG (30). Both inhibitors were effective in reducing the exATP-triggered release of MVs (*p* = 0.0006 and *p* = 0.0002, respectively) but did not alter the cytokine-mediated release (Figures 5A–C). As some of the P2X7 functions can be ascribed to the opening of the associated hemichannel pannexin-1, the effect on MV release of two of its selective inhibitors, probenecid (31) and the small peptide ^10^Panx1 (32), was checked. Also in this case, no reduction was observed in the number of MVs released by cells treated with cytokines in the presence of the pannexin-1 inhibitors (Figures 5D–F). According to a previous study (7), the activation of the lipid-metabolizing enzyme acid sphingomyelinase (ASMase) is a crucial step for the formation of MVs due to the generation of ceramide and phosphorylcoline from sphingomyelin. We chose imipramine and siramesine, two structurally unrelated ASMase antagonists (33), to verify the contribution of this enzyme on MV release mediated by IFN-γ and IL-4. The two drugs were not effective in modulating such process (Figures 5G–I). Overall, this result suggests that, in order to induce the release of MVs, ATP, and cytokines operate along very different pathways, not only in terms of signaling activation but also, more interestingly, on the basic biochemical/structural processes underlying MV formation at the plasma membrane. Additionally, considering that (i) the kinetics of the two processes are so different in time (minutes versus several hours) and (ii) many effects of cytokines are mediated by activation of transcriptional factors, we sought to determine whether inhibition of transcription could distinguish the two systems.

### Cytokine-Mediated MV Release Is Dependent on Transcription

**Figure 6:**
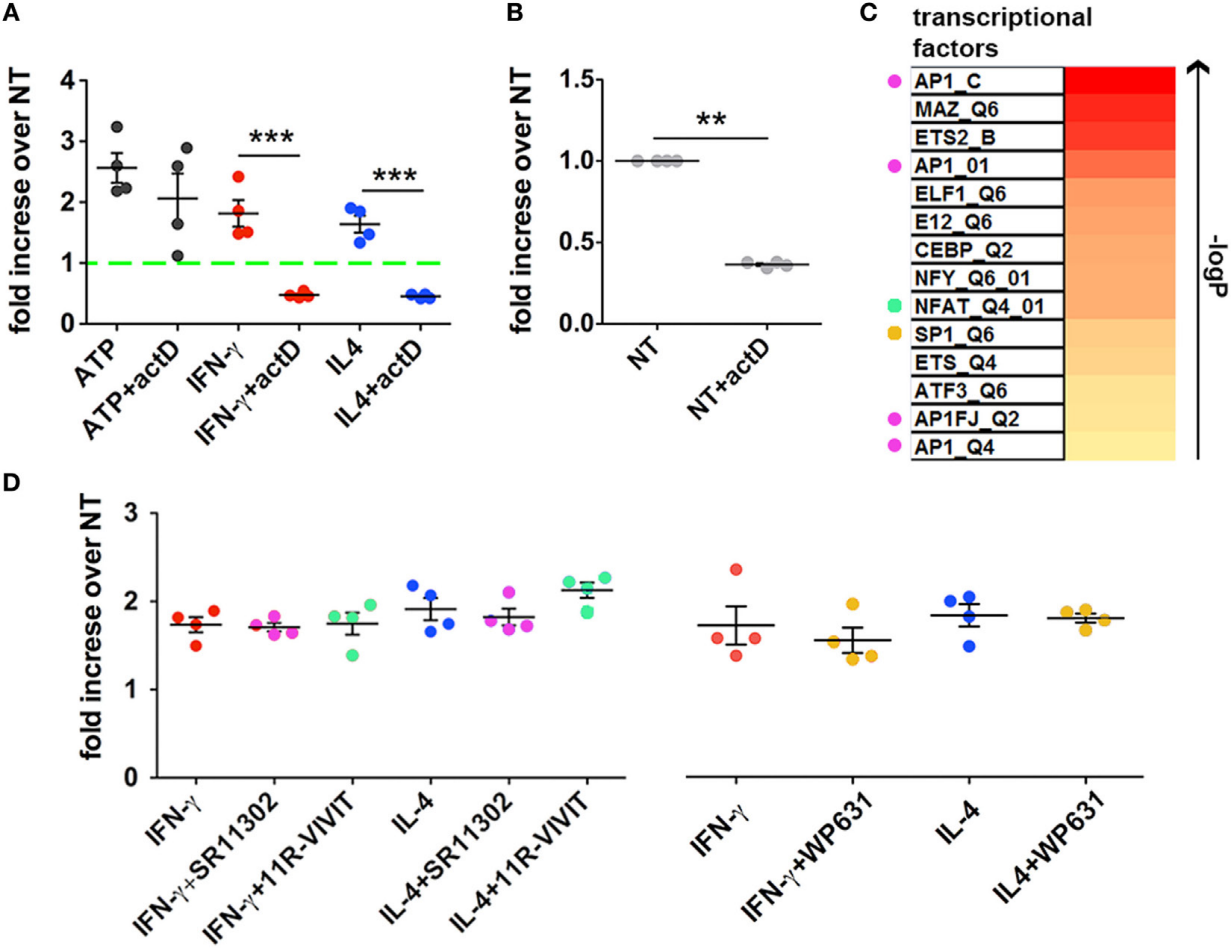
Inhibition of transcription blocks cytokine- but not ATP-mediated Microvesicle (MV) release. **(A)** CHME-5 cells were cultured in the presence or absence of actinomycin D (actD) (5 ng ml^−1^) starting 1 h before and during interferon (IFN)-γ or interleukin-4 (IL-4) treatments (20 ng ml^−1^, 24 h); ATP (1 mM, 30 min) was given to cells pretreated for 24 h with actD (ATP + actD on the graph) or not (ATP on the graph). MVs were then collected, stained with IB4-FITC, and analyzed by flow cytometry. Data are represented as fold increase compared to the untreated controls (mean ± SEM of four independent experiments). actD significantly reduced the release of MVs induced by cytokines but not that induced by ATP. Unpaired two-tailed Student’s *t*-test was used for statistical analyses (***p: 0.0009/*p*: 0.0002, respectively). **(B)** The release of MVs from cells treated with actD only was evaluated as in panel **(A)**. Data are represented as fold increase compared to the control (not treated, NT on the graph) (mean ± SEM of four independent experiments). actD significantly reduced the “basal release” of MVs. Unpaired two-tailed Student’s *t*-test was used for statistical analyses (**p: 0.0086). **(C)** The heatmap ranks the transcriptional factors based on the strength of association with the transcripts co-regulated by IFN-γ and IL-4 treatments. Colored dots indicate the transcriptional factors that can be pharmacologically modulated. **(D)** CHME-5 cells were treated with cytokines (20 ng ml^−1^) for 24 h in the presence or absence of the AP-1 antagonist SR11302 (1 µM), the NFAT blocking peptide 11 R-VIVIT (1 µM) and the SP-1 inhibitor WP631 (100 nM); MVs were then analyzed by flow cytometry. Data are represented as fold increase compared to the untreated controls (mean ± SEM of four independent experiments). The color code used in the graph for the inhibitors is the same applied in panel **(C)** for labeling the corresponding targets. One-way ANOVA plus Dunnet’s *post hoc* test was used for analyzing the effects of SR11302 and 11R-VIVIT on cytokine-mediated MV release, while unpaired two-tailed Student’s *t*-test was used for WP631.

For this purpose, CHME-5 cells were cultured for 24 h in the presence of actD, a potent inhibitor of transcription, before cytokine or ATP treatment. Interestingly, actD completely blocked the effect of the two cytokines (*p* = 0.0009 and *p* = 0.0002) but not of exATP on MV production (Figure 6A). Moreover, the number of MVs released by cytokine-treated cells in the presence of the inhibitor was significantly lower than the number of MVs released by untreated cells. Indeed, cells treated with just actD released much less vesicles with respect to the unstimulated controls (*p*: 0.0086) (Figure 6B).

According to these data, it seems that also the constitutive release of MVs, i.e., that occurs without any stimulations, is dependent on the transcriptional activity of the cell.

In order to better define such process we looked into a transcriptomic dataset, already deposited by us (10), of macrophages treated with IFN-γ or IL-4. Common regulated transcripts were identified and the enrichment in specific transcription factor/s in the selected dataset was predicted by bioinformatical annotation. Based on the FDR-corrected *p*-value of the hypergeometric probability, we found that at least eleven TFs were strongly associated with the most representative upregulated transcripts; a list of such TFs is reported in the heatmap of Figure 6C. Since some of them, such as AP-1, NFAT, and SP-1, can be pharmacologically modulated (34–36), we treated CHME-5 cells with their respective inhibitors before IFN-γ and IL-4 stimulation: however, none of them was able to reduce the amount of MVs released after cytokine treatment (Figure 6D). These results exclude the involvement of AP-1, NFAT, and SP-1 in regulating the cytokine-mediated release of MVs and will restrict the next interventions to the other TFs we reported to be strongly associated to both IFN-γ and IL-4 signaling.

## Discussion

Previous studies had already demonstrated that during neuroinflammation myeloid cells release MVs to body fluids, possibly as a simple consequence of cell degeneration or as part of the pathogenetic mechanisms involved in this process (6). However, nothing was known about the agents responsible for this response and about the mechanisms that mediate this vesicle release.

Extracellular ATP operates as a strong enhancer of membrane blebbing and shedding upon activation of its receptor P2X7, a low-affinity ion channel widely expressed in the nervous and immune system. For this reason, exATP is widely recognized as an important candidate for explaining the above observation. In the nervous system exATP can be found after secretion by microglia cells (27, 37, 38), after release from astrocytes through the activated P2X7 and/or pannexin-1 (38, 39), or after cell death (40). Among the known receptors of purines, only P2X7 has been recognized to sustain the generation of MVs (13). Compared to the other members of the P2X family, such as P2X1 and P2X3 (EC_50_ = 10 µM), P2X7 has the lowest affinity for ATP (EC_50_ = 500 µM) (41). Therefore, high amounts of exATP are needed to activate P2X7 and trigger the production of MVs. Indeed ATP, abundantly released from degenerating cells during inflammation, is assumed to be rapidly transformed into ADP, AMP and adenosine by efficient cell surface-resident enzymes, known as ecto-ATPases (such as CD39 and CD73). These changes may reduce the ability of exATP to activate the P2X7 receptor in chronic inflammation (42).

Cytokines are the best recognized mediators able to induce and orchestrate the many different aspects underlying immune responses. In our investigation we reported that MV release is stimulated by numerous cytokines related to inflammation: IFN-γ and IL-4, studied in more detail, and also IL-13, IL-27, IL-23, and TGF-β. The unexpected result was the very similar effect on MV release induced by the first two cytokines, which induce opposite effect in inflammation: stimulation by IFN-γ and inhibition by IL-4. Therefore, the stimulation of MV release might be not a direct consequence to inflammation *per se*, since it is also involved in the mechanisms acting in its resolution. Enhanced MV production in response to pro-regenerative or inflammatory cytokines may rather reflect the increased capacity of reactive microglia to communicate with the microenviroment. This is in line with the fact that, by intricate feedback loops, pro- and anti-inflammatory cytokines regulate their own expression as well as the activity of the antagonist cytokines balancing and stabilizing inflammatory reactions (43). During chronic inflammation, such as that occurring in neurological disorders like multiple sclerosis, it is likely that the two types of cytokines play coordinated effects.

The previously reported mitogenic properties of IL-4 (24) were not observed in our cell systems. Actually, in agreement with previous observations (21, 23), significant anti-proliferative effects were observed for IFN-γ and, although to a lesser extent, IL-4. With further experiments we revealed that cytokine-induced shedding is fully different from all steps of the ATP/P2X7-R signaling axis: in fact, antagonists of the receptor such as oxATP and BBG did not reduce MV numbers. The involvement of pannexin-1, the hemichannel associated to P2X7, was excluded by probenecid treatment as well as by using a more specific inhibitor, a synthetic blocking peptide.

Neither imipramine nor siramesine, two ASMase inhibitors previously reported to block the response to exATP (7, 33), were effective. These results exclude that the MV release mediated by IFN-γ and IL-4 depends on a pathway analogous to that responsible for the exATP-mediated shedding, which is acutely enhanced upon P2X7 activation.

The dynamics of ATP- and cytokine-mediated shedding are very different (few minutes versus 24 h, respectively), making also a quantitative comparison difficult, although cytokines in general appear less potent than ATP. The relatively long time required by cytokines made us hypothesize that their ability to induce MV release is related to the transcription of genes controlled by the same cytokines. A subtoxic concentration of actD, a potent inhibitor of transcription, completely abolished MV production in the presence of cytokines but not of ATP. Even more interestingly, in the presence of the inhibitor the number of MVs produced was lower than that of untreated cells. The latter differential results obtained with cytokines and ATP strongly suggest that: (i) the factor/s involved in cytokine-enhanced MV generation is/are the same required for the constitutive MV release and, as a consequence (ii) the promoting effects of cytokines on MV release may represent an increase of the basal shedding activity present at the plasma membrane of all cells. In contrast, the antagonists of P2X7 signaling pathway did not reduce significantly the constitutive release of MVs, as previously described (7). Since cytokines induce transcription of specific gene networks, future work will be aimed at the identification of the genes involved in vesicle generation. In our hands, the inhibition of three out of the eleven transcriptional factors we found to be strongly associated to the genes regulated by both IFN-γ and IL-4 were ineffective in reducing the release of MV after cytokine treatment. In order to clarify the contribution of exATP in the generation of the myeloid cell-derived MVs released during neuroinflammation, we extended the work to a disease model. Mice affected by EAE, a model of human multiple sclerosis, were treated with imipramine, which changed neither the amount of MVs released to the CSF in the chronic phase of the disease, nor the neurological traits of the diseased animals. Therefore, it seems that an ASMase-independent mechanism is responsible for the enhanced release of MVs in mice with EAE, perhaps controlled by cytokine signaling. Although this conclusion is encouraging, two considerations about the role of exATP should be clarified: (i) cytokines and purinergic signaling are linked by the fact that the P2X7 receptor is upregulated by the former as observed by us and others (44–46). This may be indicative of the fact that cytokines-primed cells are somehow sensitized to the exATP released by surrounding cells; whether this upregulation is sufficient to contribute to plasma membrane shedding *in vivo* has to be proven. In addition, agonists of TRPV1channels have been also recently shown to acutely enhance MV production from microglia through a pathway involving p38 MAP kinase, an enzyme which is also activated downstream P2X7 (47). (ii) The role of P2X7-dependent signaling in EAE is controversial: in fact, treating EAE mice with oxATP or BBG reduced the severity of the disease, with a decrease of neuronal loss and increase of oligodendroglia survival (45), whereas P2X7 knockout mice displayed a more severe pathology (48). However, another study based on a similar knockout model reported opposite results (49). Anyway, whether the positive role of P2X7 antagonists on the pathology depends on modulation of MV release, is not known.

Our results obtained in cell lines *in vitro*, not necessarily reflect what happens *in vivo*. Further, cell lines are transformed cells similar to tumor cells, in which EVs release has been associated to their biological features (i.e., malignant/benign) (50), suggesting even more caution in interpretation of data. Nevertheless, we reproduced our results also in primary cells, and pannexin-1 knockout mice develop a milder form of EAE (51). We showed, however, that pannexin-1 inhibition *in vitro* did not result in a reduction of ATP-induced MV release. Hence, in this case, the amelioration of the disease cannot be related to a variation of the MV number due to a modulation of exATP-related signaling.

Another important step to establish the contribution of the two systems to MV release during inflammation will derive from the understanding of the molecular factor/s controlling the cytokine-induced shedding. However, since such factors are likely to be involved in the MV release responses induced by both pro- and anti-inflammatory cytokines, the consequences of their modulation in the animal model of neuroinflammation are hard to predict.

The possibility, suggested by our results, that constitutive and cytokine-induced membrane shedding share molecular pathways, indicates a new path to investigate vesicle biogenesis and suggests new tools to control genetically or pharmacologically MV release.

## Conclusion

The generation and release of EVs is one of the fields that attract greatest attention in the current cell biology research. Among the various areas of this research those that are becoming more and more important are those dealing with diseases in which EVs are recognized to play significant roles. In contrast, the study of the mechanisms that sustain the generation and release of EVs receive much less attention, mainly as far as MVs are concerned. As a consequence, these mechanisms remain largely unclear.

In our work, we have considered such a problem under different perspectives. Inflammation had already been intensely investigated as a pathological condition in which EV release is involved; indeed many cell types involved in inflammation are highly competent in EV release. Thus, the release was largely believed to be a process important in this context. This work, carried out by using microglia cell lines, has demonstrated that not only both pro- and anti-inflammatory cytokines induce the release of MVs but also that this process is distinct from the one acutely mediated by the ATP/P2X7 pathway. Another important finding about the EV release induced by cytokines is its dependence to transcription. This property, however, needs further studies before the mechanism is clarified *via* the identification of the specific genes that are involved.

## Ethics Statement

All procedures involving animals were performed according to the animal protocol guidelines prescribed by the Institutional Animal Care and Use Committee (IACUC) authorization no. 643 at San Raffaele Scientific Institute (Milan, Italy).

## Author Contributions

FC, CV, and RF designed and conceived the study. FC performed most of the experiments; FC, MB, and AN performed cytofluorimetric measurements and analyzed the data. PP prepared the samples for electron microscopy and acquired the images; FC, AF, and GC carried out the animal experiments. MR and FC analyzed transcriptomic data. CF and RF wrote the paper. All authors revised and approved the draft.

## Conflict of Interest Statement

The authors declare that the research was conducted in the absence of any commercial or financial relationships that could be construed as a potential conflict of interest. The reviewer PI and handling editor declared their shared affiliation.

## Supplementary Material

Figure S1 Expression and characterization of P2X7 in CHME-5 cells. The expression of the P2X7 receptor in CHME-5 cells at both mRNA and protein level was assessed by RT-PCR **(A)** and western blotting **(B)**, respectively. HEK-293T cells were used as negative controls. The data shown in **(A,B)** are representative of two independent experiments. **(C)** The functionality of the receptor in CHME-5 cells has been proven by fluorescence. Ethidium bromide uptake was tested in the presence or absence of ATP (1 mM, 20 min); only in the former condition the ethidium fluorescence appears inside the cells. **(D)** ATP-mediated Ca^2+^ influx in CHME-5 cells was assessed by imaging; cells were pre-loaded with the Ca ^2+^ indicator Fluo-4AM (4 mM) and treated or not with ATP (1 mM); ATP-treated cells (right panel) are significantly brighter than those left untreated (left panel). Full-length blots are presented in Figure S4 in Supplementary Material.

Figure S2 Purification, quantification, and characterization of microvesicles (MVs) from CHME-5 cells. **(A)** Description of the differential ultracentrifugation protocol applied for extracellular vesicle (EV) purification. **(B)** A representative negative-stained pellet resulting from the 10,000 *g* centrifugation step; the picture on the right is a magnification of the first. Both scale bars are of 500 nm. The graph represents the vesicle diameters plotted against their frequency over the total values. Dynamic light scattering **(C)** and tuneable resistive pulse sensing **(D)** analyses of the resuspended 10,000 *g* pellet. **(E)** EVs and whole cell protein content was assessed by western blotting; after immunoblotting for flotillin-1, the filter was stripped and incubated with an anti-CD63 antibody. Full-length blots are presented in Figure S4 in Supplementary Material.

Figure S3 Interferon (IFN)-γ and interleukin-4 (IL-4) reduce microglia cell proliferation. **(A,D)** Percentage of Ki67^+^ BV2 cells **(A)** and CHME-5 cells **(D)** after exposure to cytokines (20 ng ml^−1^) for the indicated time length; **(B,E)** the total BV2 **(B)** and CHME-5 **(E)** cell number, quantified by DAPI staining, refers to the 24 h cytokine treatments. **(C,F)** MTT assay for measuring BV2 **(C)** and CHME-5 **(F)** cell vitality 24 h after treatments. One-way Anova plus Dunnett’s *post hoc* test were used for statistical analyses (*p*-values are indicated over the bars). The results are shown as the mean ± SEM of three independent experiments. **(G)** Apoptosis has been assayed by western blotting for the activated form of caspase-3 in lysates of CHME-5 cells treated with staurosporin (1 mM, 24 h), ATP (1 mM, 30 min), and cytokines (20 ng ml^−1^, 24 h). **(H)** The presence of IL-1b inside microvesicles (MVs) produced by different stimuli was measured by ELISA. MVs were purified by differential ultracentrifugation from supernatants of CHME-5 cells left untreated or stimulated with lipopolysaccaride (LPS) (100 ng ml^−1^, 24 h), LPS (100 ng ml^−1^, 24 h) + ATP (1 mM, 30 min), interferon (IFN)-γ, interleukin-4 (IL-4) (20 ng ml^−1^, 24 h). IL-1β was detected associated to MVs produced after LPS and ATP but not cytokine treatments. The graph represents the mean concentrations of IL-1-β ± SEM of two independent experiments. Full-length blots are presented in Figure S4 in Supplementary Material.

Figure S4 Uncropped films of the immunoblots reported in the main-text figures are shown. Red boxes indicate the area of the film shown in the corresponding figure (as indicated in the heading).

video S1 CHME-5 cells respond to ATP by plasma membrane blebbing and shedding. CHME-5 cells, seeded on glass coverslips, were transfected with farnesyl-GFPa and imaged in the presence of ATP (1mM,7 minutes). Eight frames of the video (from 02:30 and 02:45 minutes after ATP addition) are reported in Figure 2.
