# Supplementary figures and images for: Cytokines Stimulate the Release of Microvesicles from Myeloid Cells Independently from the P2X7 Receptor/Acid Sphingomyelinase Pathway
Source: Front Immunol. 2018 Feb 7;9:204. doi: 10.3389/fimmu.2018.00204 (PMC5808348; doi:10.3389/fimmu.2018.00204)

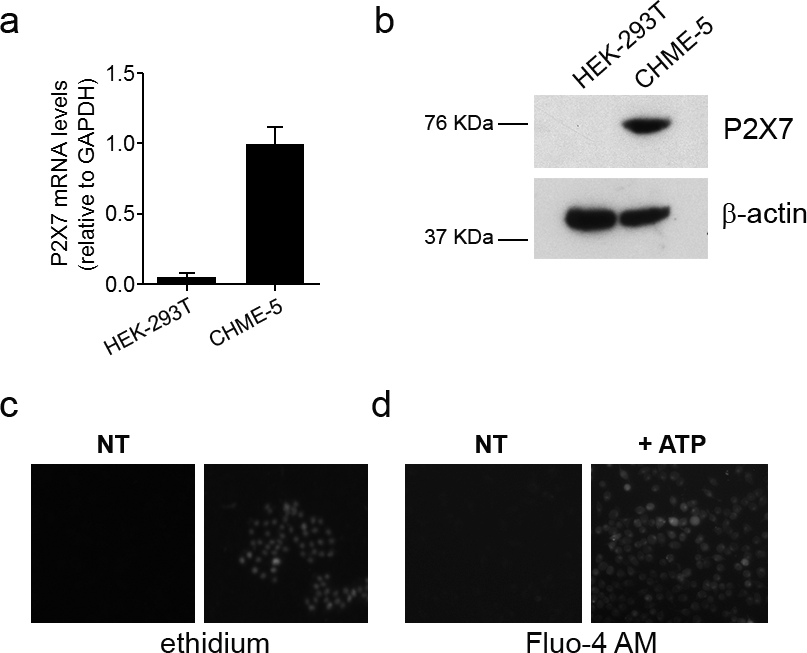

Supplement: Figure S1 — Expression and characterization of P2X7 in CHME-5 cells. The expression of the P2X7 receptor in CHME-5 cells at both mRNA and protein level was assessed by RT-PCR (A) and western blotting (B), respectively. HEK-293T cells were used as negative controls. The data shown in (A,B) are representative of two independent experiments. (C) The functionality of the receptor in CHME-5 cells has been proven by fluorescence. Ethidium bromide uptake was tested in the presence or absence of ATP (1 mM, 20 min); only in the former condition the ethidium fluorescence appears inside the cells. (D) ATP-mediated Ca2+ influx in CHME-5 cells was assessed by imaging; cells were pre-loaded with the Ca 2+ indicator Fluo-4AM (4 mM) and treated or not with ATP (1 mM); ATP-treated cells (right panel) are significantly brighter than those left untreated (left panel). Full-length blots are presented in Figure S4 in Supplementary Material. [file Image_1.tif]

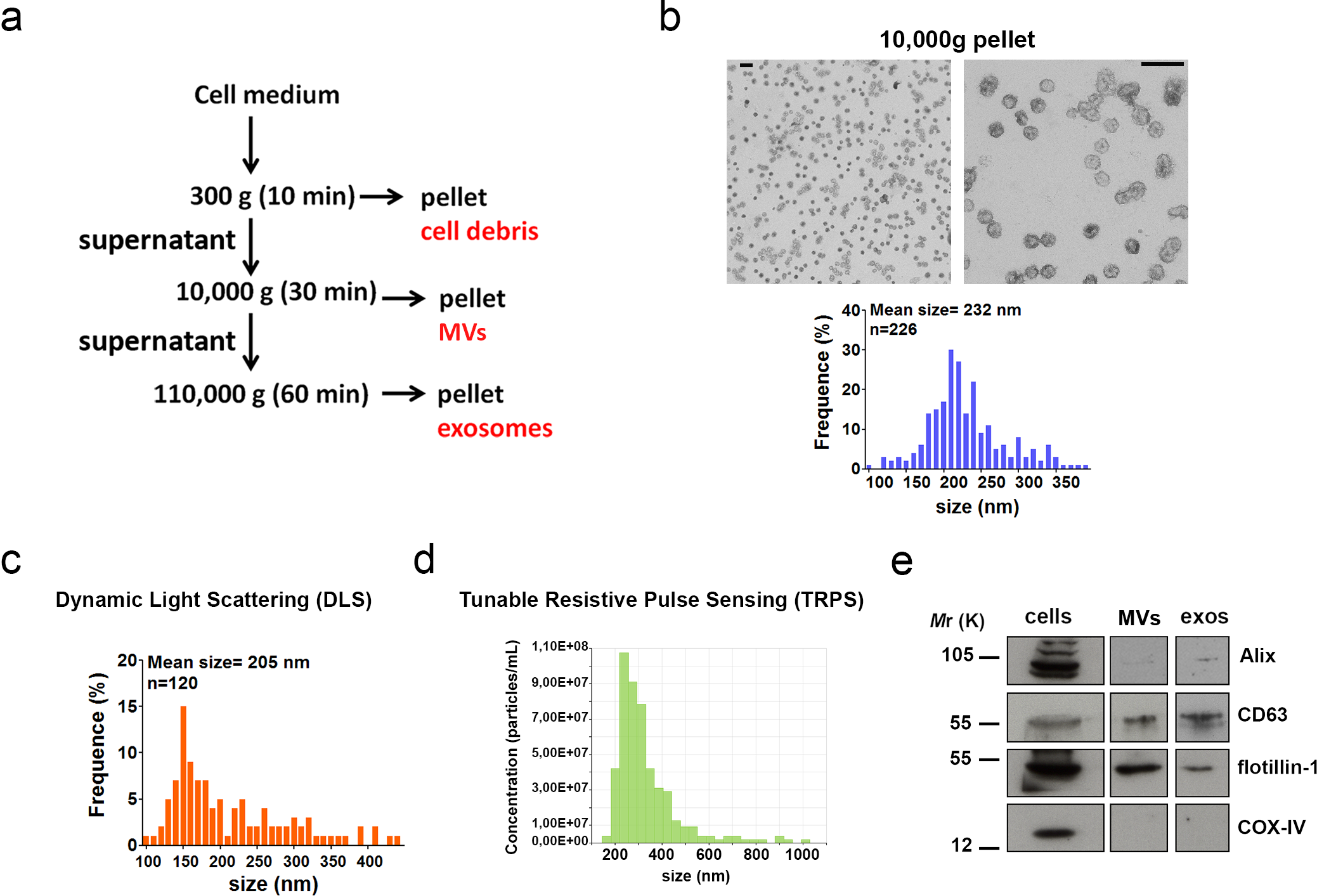

Supplement: Figure S2 — Purification, quantification, and characterization of microvesicles (MVs) from CHME-5 cells. (A) Description of the differential ultracentrifugation protocol applied for extracellular vesicle (EV) purification. (B) A representative negative-stained pellet resulting from the 10,000 g centrifugation step; the picture on the right is a magnification of the first. Both scale bars are of 500 nm. The graph represents the vesicle diameters plotted against their frequency over the total values. Dynamic light scattering (C) and tuneable resistive pulse sensing (D) analyses of the resuspended 10,000 g pellet. (E) EVs and whole cell protein content was assessed by western blotting; after immunoblotting for flotillin-1, the filter was stripped and incubated with an anti-CD63 antibody. Full-length blots are presented in Figure S4 in Supplementary Material. [file Image_2.tif]

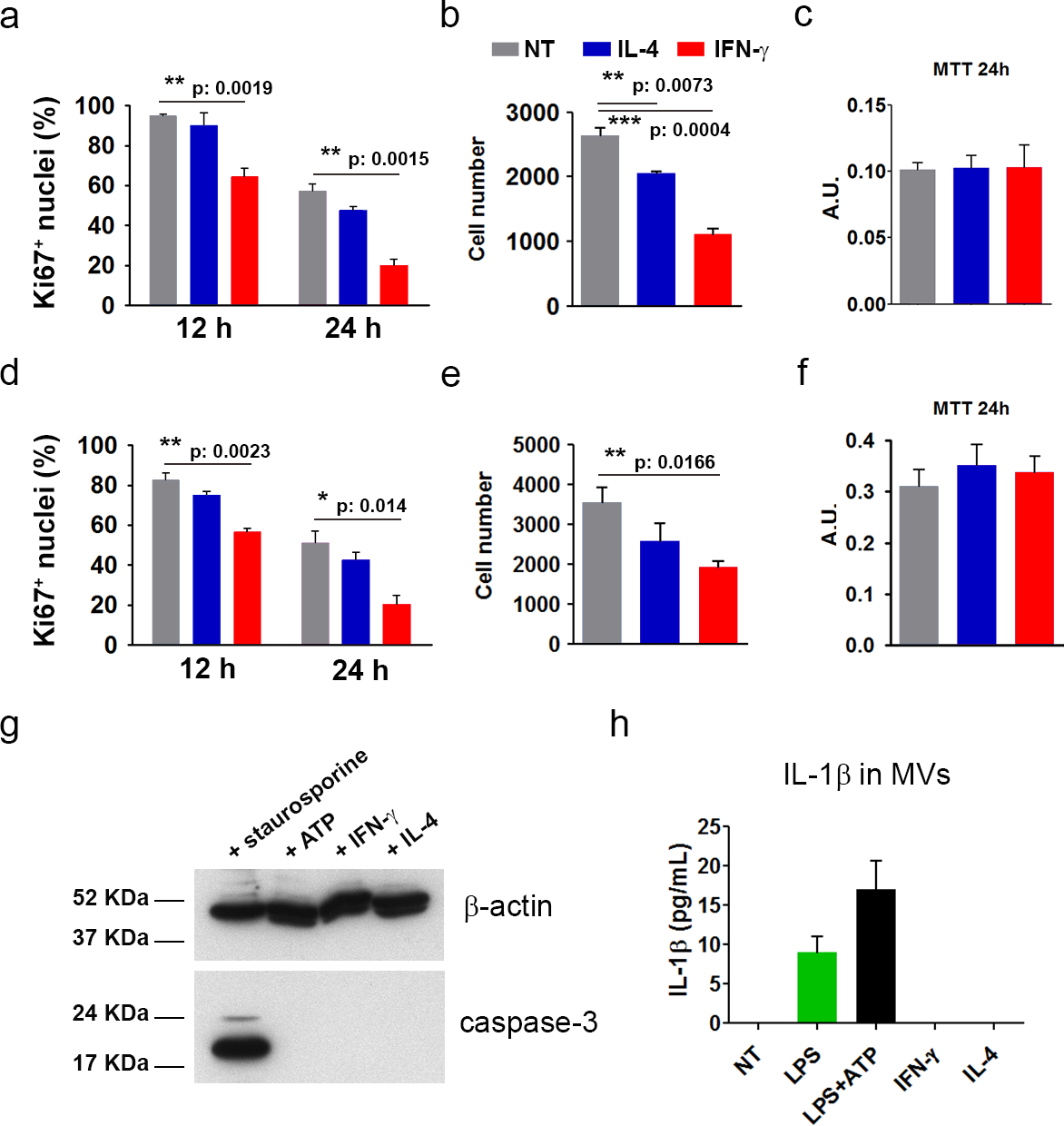

Supplement: Figure S3 — Interferon (IFN)-γ and interleukin-4 (IL-4) reduce microglia cell proliferation. (A,D) Percentage of Ki67+ BV2 cells (A) and CHME-5 cells (D) after exposure to cytokines (20 ng ml−1) for the indicated time length; (B,E) the total BV2 (B) and CHME-5 (E) cell number, quantified by DAPI staining, refers to the 24 h cytokine treatments. (C,F) MTT assay for measuring BV2 (C) and CHME-5 (F) cell vitality 24 h after treatments. One-way Anova plus Dunnett’s post hoc test were used for statistical analyses (p-values are indicated over the bars). The results are shown as the mean ± SEM of three independent experiments. (G) Apoptosis has been assayed by western blotting for the activated form of caspase-3 in lysates of CHME-5 cells treated with staurosporin (1 mM, 24 h), ATP (1 mM, 30 min), and cytokines (20 ng ml−1, 24 h). (H) The presence of IL-1b inside microvesicles (MVs) produced by different stimuli was measured by ELISA. MVs were purified by differential ultracentrifugation from supernatants of CHME-5 cells left untreated or stimulated with lipopolysaccaride (LPS) (100 ng ml−1, 24 h), LPS (100 ng ml−1, 24 h) + ATP (1 mM, 30 min), interferon (IFN)-γ, interleukin-4 (IL-4) (20 ng ml−1, 24 h). IL-1β was detected associated to MVs produced after LPS and ATP but not cytokine treatments. The graph represents the mean concentrations of IL-1-β ± SEM of two independent experiments. Full-length blots are presented in Figure S4 in Supplementary Material. [file Image_3.tif]

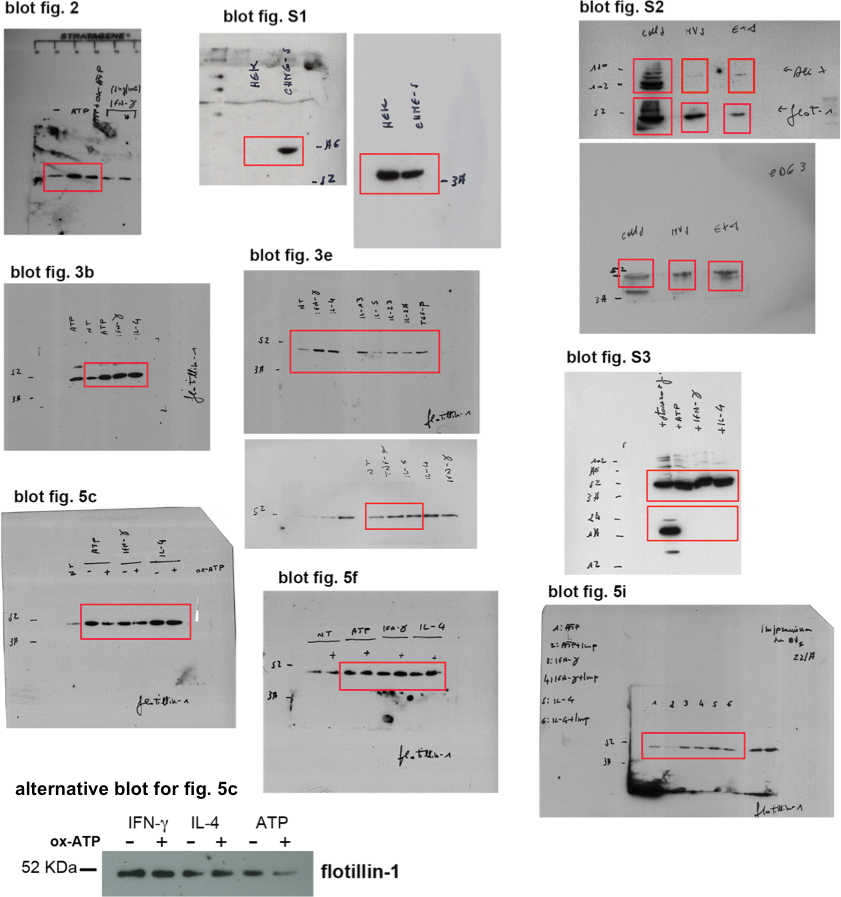

Supplement: Figure S4 — Uncropped films of the immunoblots reported in the main-text figures are shown. Red boxes indicate the area of the film shown in the corresponding figure (as indicated in the heading). [file Image_4.tif]
